# First Assembly of a Draft Genome of the Critically Endangered Northern Muriqui ( *Brachyteles hypoxanthus* , Primates, Atelidae) Including Non‐Invasive Genotyping Strategies for the Species

**DOI:** 10.1002/ece3.71356

**Published:** 2025-08-19

**Authors:** Amanda Alves de Melo‐Ximenes, Romina Batista, Leonardo Carlos Jeronimo Corvalán, Tomas Marques‐Bonet, Lukas Kuderna, Kyle Farh, Jeffrey Rogers, Mariane da Cruz Kaizer, Jean Philippe Boubli, Fabiano Rodrigues de Melo, Rhewter Nunes, Mariana Pires de Campos Telles

**Affiliations:** ^1^ Laboratório de Genética & Biodiversidade Instituto de Ciências Biológicas I, Universidade Federal de Goiás Goiânia Goiás Brazil; ^2^ Instituto Nacional de Pesquisas da Amazônia, INPA Manaus Amazonas Brazil; ^3^ School of Science, Engineering and the Environment, University of Salford Salford England UK; ^4^ Department of Biological and Environmental Sciences University of Gothenburg Gothenburg Sweden; ^5^ Gothenburg Global Biodiversity Centre University of Gothenburg Gothenburg Sweden; ^6^ Laboratório de Bioinformática e Biodiversidade (LBB), Instituto Acadêmico de Ciências da Saúde e Biológicas (IACSB) Universidade Estadual de Goiás – Campus Oeste – UnU de Iporá Iporá Goiás Brazil; ^7^ Institut de Biologia Evolutiva, Universitat Pompeu Fabra Barcelona Spain; ^8^ Illumina Inc. San Diego California USA; ^9^ Baylor College of Medicine Houston Texas USA; ^10^ Instituto Nacional da Mata Atlântica Santa Teresa Espírito Santo Brazil; ^11^ Rede Eco‐Diversa Para Conservação da Biodiversidade Tombos Minas Gerais Brazil; ^12^ Departamento de Engenharia Florestal Universidade Federal de Viçosa Viçosa Minas Gerais Brazil; ^13^ Escola de Ciências Médicas e da Vida Pontifícia Universidade Católica de Goiás Goiânia Goiás Brazil

**Keywords:** conservation genomics, GBS, genomics, HTS, SSR, SSR‐Seq

## Abstract

Genomic resources, such as draft genomes, are vital for biodiversity monitoring and conservation. For endangered species, they enable the development of tools like organellar genomes and molecular markers, which are crucial for population genetics. Advances in sequencing technologies now allow high‐throughput genotyping with detailed amplicon sequences, enhancing genetic variation studies. The northern muriqui ( *Brachyteles hypoxanthus* ), a critically endangered primate endemic to Brazil's Atlantic Forest, currently lacks both nuclear and mitochondrial genome data and species‐specific microsatellite markers for population genetic studies. We assembled a 2.52 Gb draft genome for *B. hypoxanthus* with 202,243 contigs (N50 = 29,134 bp), and BUSCO analyses indicated 52% completeness and 15.5% fragmented genes. The complete 16,635 bp mitochondrial genome retains the conserved mammalian structure with 22 tRNAs, 2 rRNAs, 13 CDS, and an origin of replication. Additionally, we designed 31 SSR primer pairs suitable for non‐invasive sampling and genotyping, alongside two mtDNA and two sex‐determination primers, configured into three multiplex PCR sets. These genomic resources, including the draft genome, complete mitochondrial genome, and microsatellite markers, provide essential tools for evolutionary analyses and the genetic monitoring of *B. hypoxanthus* populations, supporting its conservation.

## Graphical abstract

First draft genome assembly of the critically endangered primate northern muriqui ( *Brachyteles hypoxanthus* ) and complete mitochondrial genome assembly. Set of microsatellite markers developed for the species for genotyping‐by‐sequencing using high‐throughput sequencing technology and aiming to amplify non‐invasive sampling for population genetics purposes.

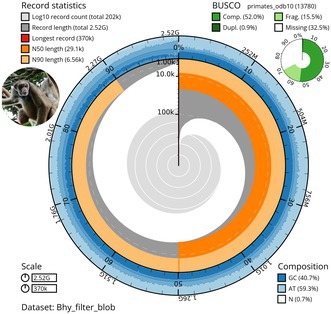

## 1 Introduction

The current rate of biodiversity loss, threatened by habitat degradation and climate change, indicates a trajectory of declining population and species diversity (Ceballos et al. 2020; Cowie et al. 2022). This is true for the Atlantic rain forest of Brazil, a hotspot of biodiversity (Myers et al. 2000), that remains with only 13% of its Pre‐Columbian coverage (Lins‐e‐Silva et al. 2021; Oliveira‐Filho and Fontes 2000). Efforts to prevent species extinction and population decline require not only mitigation efforts such as habitat restoration but also research on species ecology, biology, and genetics (Brandies et al. 2019). An important component of population viability is the long‐term maintenance of genetic diversity at all levels of biological organization (Theissinger et al. 2023). Genomic information can provide tools to characterize biodiversity and support conservation efforts aimed at preserving genetic diversity by mapping genomic variation, crucial information for management of endangered species (Formenti et al. 2022; Theissinger et al. 2023).

The generation of genomic resources, such as draft and reference genomes, can aid in conservation issues such as taxonomic identification, biodiversity monitoring and management, identification of biodiversity hotspots for conservation prioritization, and estimation of genetic diversity, structure, and demographic history (Supple and Shapiro 2018; Theissinger et al. 2023). These applications can be achieved through a variety of methodological techniques, including DNA barcoding and metabarcoding, reduced genome representation, RNA sequencing, and whole genome sequencing (Brandies et al. 2019; Theissinger et al. 2023). Achieving complete reference genomes for vertebrates, especially mammals, is unlikely to be achieved with a single sequencing technology such as short‐read sequencing, but draft genomes obtained from short‐read sequencing can answer many conservation questions and aid in the assembly and development of other genomic and genetic tools, such as organellar genomes and molecular markers (Fuentes‐Pardo and Ruzzante 2017; Gandomkar et al. 2021; Zhou et al. 2014).

Molecular markers that are highly polymorphic, such as microsatellites, are useful for genetically distinguishing individuals and are therefore widely used in population and conservation genetics and forensic studies (Bruford and Wayne 1993; Guichoux et al. 2011; Hodel et al. 2016). Applications in wildlife forensics include parentage analysis, identification of geographic origin, and counting individuals in illegal hunts (Ng et al. 2017; Ogden and Linacre 2015; Sanches et al. 2012). Additionally, in population and conservation genetics, microsatellites remain excellent markers of choice for genetically characterizing wild populations and studying their degree of fragmentation, dispersal patterns, mating systems, relatedness among individuals, and levels of inbreeding (Kleinhans and Willows‐Munro 2019; Mantellatto et al. 2017; Moran et al. 2021; Oklander et al. 2017; Pinho et al. 2014; Strier et al. 2011).

Microsatellites are also particularly valuable when working with non‐invasive samples, which are an important source of biological material for wildlife monitoring but are often insufficient for complex genomic methods that require high‐quality DNA, such as genome resequencing (Brandies et al. 2019). Much genetic information about wild populations can be assessed through non‐invasive sampling, such as rates of gene flow between populations and estimates of inbreeding (Arandjelovic et al. 2011; Pinho et al. 2014), which helps to understand how genetic diversity is distributed across geographic locations and which conservation strategies are more appropriate to maintain sustainable species (Ortega et al. 2020).

Working with degraded DNA samples obtained from non‐invasive primate sampling, such as feces and hair, is a real challenge for all types of molecular markers and their different assessment methods (Carroll et al. 2018; Taberlet et al. 1996). However, there are some strategies that can be applied to overcome the difficulties of obtaining reliable genotypes from low‐quality DNA samples, ranging from target enrichment (Carroll et al. 2018) to the development of personalized markers and multiple PCR replicates (Trede et al. 2021). In addition, the use of non‐invasive sampling reduces contact between wildlife and humans, reducing animal stress and the potential for disease transmission.

With the advances in high‐throughput sequencing technologies, it has become possible to genotype hundreds of individuals for dozens of microsatellite loci with the complete amplicon sequence available, providing more information on genetic variation (de Barba et al. 2017; Donaldson et al. 2020; Nishio et al. 2021; Pimentel et al. 2018; Vartia et al. 2015). The strategy of genotyping microsatellite loci by sequencing, also known as simple sequence repeat sequencing (SSR‐Seq), overcomes the difficulties and drawbacks of traditional SSR genotyping‐by‐capillary‐electrophoresis, such as fragment size homoplasy, delayed fragment migration, and low interlaboratory comparability (de Barba et al. 2017; Guichoux et al. 2011).

When developing SSR markers for genotyping‐by‐sequencing (GBS), the size of the sequencing read, and therefore, the amplicon size must be taken into account when using short‐read platforms (Lepais et al. 2020; Vartia et al. 2015). Additionally, when working with DNA from non‐invasive samples to amplify microsatellite loci, it is important to consider specific precautions during primer design to minimize common artifacts, such as stutter and allele dropout, which can occur due to low DNA quality and quantity. These precautions include choosing long motif sizes and small allele sizes (de Barba et al. 2017; Trede et al. 2021).

Primates have been the subject of studies to develop new microsatellite markers for GBS and to test their applicability for non‐invasive sampling of wild populations, where reliable genotypes could be obtained and new genetic variation could be detected due to access to the whole amplicon sequence (Barbian et al. 2018; Trede et al. 2021). The northern muriqui ( *Brachyteles hypoxanthus* Kuhl, 1820) is a critically endangered primate species endemic to the Atlantic forest of Brazil, with currently 12 known populations (Strier et al. 2017). This species is an icon of the Brazilian Atlantic forest but suffers from many anthropogenic threats, such as deforestation and zoonotic disease (Strier et al. 2019; Valença‐Montenegro et al. 2021). With the increasing isolation of northern muriqui populations due to habitat fragmentation, management actions are needed to ensure the survival of the species, and information on the genetic structure, variability, and inbreeding of these populations is helpful in defining sustainable management actions (Frankham et al. 2017).

The only studies with nuclear microsatellite loci available for *B. hypoxanthus* have focused on paternity analysis and mating preferences using markers transferred from other primate species (Chaves et al. 2023; Strier et al. 2011). To date, no complete nuclear or mitochondrial genome is available for the northern muriqui, and consequently, no microsatellite markers have been developed for this species. Therefore, the main objective of this work was to sequence and assemble a draft nuclear genome, assemble a mitochondrial genome, and develop microsatellite primers for genotyping‐by‐sequencing, focusing on non‐invasive samples of the northern muriqui, *B. hypoxanthus* . To this end, we assembled a draft genome for *B. hypoxanthus* and performed a phylogenetic comparative analysis with other Atelidae genomes, searched for microsatellite regions in the northern muriqui genome, designed primers focused on SSR‐Seq, and validated these primers in silico for multiplex PCR reactions.

## 2 Materials and Methods

### 2.1 Sampling, Sequencing Genomic Processing and Assembly

We obtained a muscle tissue sample from a northern muriqui, which is deposited in the collection of the *Projeto Muriquis do Caparaó* (Minas Gerais, Brazil). Total DNA was isolated using MagAttract High Molecular Weight (Qiagen, Germany), and a genomic short‐inserted paired‐end PCR‐free library was prepared using the KAPA HyperPrep kit (Roche), with modifications (Kuderna et al. 2023). The prepared library was checked for size quality on an Agilent 2100 Bioanalyzer using the DNA 7500 assay (Agilent) and for quantity using the Kapa Library Quantification Kit (Roche). Whole genome sequencing was performed at the Institut de Biologia Evolutiva, Universitat Pompeu Fabra, Barcelona, Spain, on an Illumina NovaSeq6000 platform in 2 × 150 + 17 + 8 bp paired‐end mode.

After sequencing, raw reads were evaluated for base quality and the presence of adapters using FastQC software (Andrews 2010). Quality control and trimming were performed using Trimmomatic software (Bolger et al. 2014). The resulting high‐quality reads were selected for a de novo assembly on SPAdes v. 3.15.4 (Prjibelski et al. 2020) using the program's default settings. To improve genome assembly in terms of contiguity and completeness, we employed the following approach: we mapped raw reads to the de novo assembled genome using HISAT2 v. 2.2.1 (Kim et al. 2019), and the resulting alignment was scaffolded using BESST v. 2.0 (Sahlin et al. 2014). Assembly quality was assessed using QUAST‐LG (Mikheenko et al. 2018), and microsatellite regions were predicted using the MIcroSAtellite Identification Tool (MISA) (Thiel et al. 2003). For SSR characterization, a minimum value of ten repeats was defined for mononucleotide SSR motifs, five for dinucleotides, four for trinucleotides, and three for tetranucleotides, pentanucleotides, and hexanucleotides. To check the completeness of our newly assembled genome, we used BUSCO v.5.6.1 and its *primates_odb10* database. Contigs smaller than 500 bp were removed for BUSCO completeness assessment (Manni et al. 2021), and contigs smaller than 10,000 bp were removed for microsatellite primer development to avoid selection of microsatellite loci physically close to each other in the genome.

### 2.2 Genome Size

The genome size of *B. hypoxanthus* was estimated using KMC tools v 3.2.2 to generate a K‐mer distribution with a *K* value of 21 (Kokot et al. 2017). This distribution was used to estimate genome size and heterozygosity using GenomeScope2 (Ranallo‐Benavidez et al. 2020).

To identify potential contamination in our assembly, we plotted the blob plots (GC vs. coverage) using BlobToolKit v.4.4.0 (Challis et al. 2020). For this, the reads were aligned in the genome to estimate the coverage, and the contigs were blasted to the nucleotide non‐redundant‐NCBI database. We then removed all contigs that matched a different phylum. The BlobToolKit v.4.4.0 was also used to plot the snail plot (Challis et al. 2020). Clique ou toque aqui para inserir o texto.

### 2.3 Mitochondrial Genome Assembly and Genetic Diversity Estimation

We assembled the whole mitochondrial genome for *B. hypoxanthus* using NOVOPlasty v. 4.3.3 (Dierckxsens et al. 2017) and annotated it using the MITOS2 tool on the Galaxy online server v.2.1.8 (https://usegalaxy.eu/). Graphical visualization was performed using *Chloroplot* in R (Zheng et al. 2020). In a comparative genomic approach, we aligned the northern muriqui mitogenome to the current Atelidae species using mitogenomes available at the National Center for Biotechnology Information (NCBI) (Table S1). In addition, to validate the mitochondrial genome assembly, we estimated a phylogeny for the Atelidae family (Table S1) using a maximum likelihood approach with IQTREE v. 2.2.0 (Nguyen and Ho 2016). To determine the best‐fitting nucleotide model (TIM2 + F + R3), we used ModelFinder Plus (Kalyaanamoorthy et al. 2017) and 1000 bootstrap replicates. The species *Macaca fuscata* was used as an outgroup. The resulting phylogenetic tree was plotted using FigTree v. 1.4.4 (http://tree.bio.ed.ac.uk/software/figtree/).

We also estimated the nucleotide diversity across all species mitogenomes using DnaSP v. 5 (Librado and Rozas 2009) with a 100 bp window and a 25 bp interval. High nucleotide diversity regions were considered as 2 times the median value of nucleotide diversity () for all sites.

### 2.4 Primer Design

The identification of microsatellite regions followed by primer design was performed using QDD v.3 (Meglécz et al. 2010), as it is a complete tool that detects microsatellite regions in the context of transposable elements and therefore allows the selection of primers outside these regions to avoid the occurrence of null alleles due to primer annealing problems (Nunes et al. 2019). To reduce the amount of stuttering induced by PCR, primers were designed only for tetra‐ and pentanucleotide microsatellite motifs with a minimum of 12 and 10 repeats, respectively (de Barba et al. 2017). To increase the success of locus amplification from degraded DNA non‐invasive samples such as feces (Trede et al. 2021), we filtered to include only small‐size alleles (up to 150 bp) and consequently small amplicon sizes (from 90 to 250 bp).

Primers were designed for a minimum size of 17 bp and a maximum size of 24 bp. Melting temperature ranged from 48°C to 62°C and GC content from 35% to 50%. The Primates database of RepeatMasker was used to exclude primers within transposable elements, and we also excluded (i) microsatellite motifs with three or more consecutive adenines (AAA*) or (ii) that consisted only of AT (AT count = 100%), and (iii) primers with a minimum distance of the microsatellite region of less than 20 bp.

We used Primer3 v.4.1.0 (Untergasser et al. 2012) to design two additional primer sets to amplify mitochondrial regions of the *B. hypoxanthus* genome, using the assembled mitogenome generated in this study. To provide a means of confirming the identity of fecal samples, we designed primers for DNA barcoding using mitochondrial DNA (mtDNA). Although there are many primers available in the literature for primate DNA barcoding, we designed primers to amplify a portion of the mitogenome within the family Atelidae, which has the highest level of nucleotide diversity within its species, and to amplify shorter fragments that can be sequenced on short‐read platforms.

Previous population genetic studies of the northern muriqui have analyzed approximately 400 bp of the mitochondrial hypervariable region (HVSI) (Chaves et al. 2019, 2011; Fagundes et al. 2008). To provide the means to compare these previously sequenced mtDNA loci with future samples of *B. hypoxanthus* and *B. arachnoides* , we selected a portion of this PCR product with the highest nucleotide diversity and specific species divergent polymorphisms to differentiate the two species by sequencing on short‐read platforms. The same method of nucleotide diversity analysis using DnaSP described above was applied to the amplicons of the HVSI region of *Brachyteles* spp. available at NCBI (Table S2).

Primers previously developed for sexing Neotropical primates by non‐invasive sampling were also included in the in silico evaluation, as it may be of interest to researchers to also sex individuals sampled non‐invasively in the field (Di Fiore 2005). All primers for microsatellite and mtDNA regions and sex primers were subjected to a complete in silico evaluation regarding their physicochemical properties and PCR performance in multiplex reactions using openPrimeR image on Docker (Kreer et al. 2020). After the evaluation, the PCR multiplex composition was designed to retain only primers that, at least *in silico*, had a maximum difference of melting temperature of 5°C and no chance of forming primer cross‐ and self‐dimers (cross‐ and self‐dimer energy < −5 kcal/mol).

## 3 Results and Discussion

**FIGURE 1:**
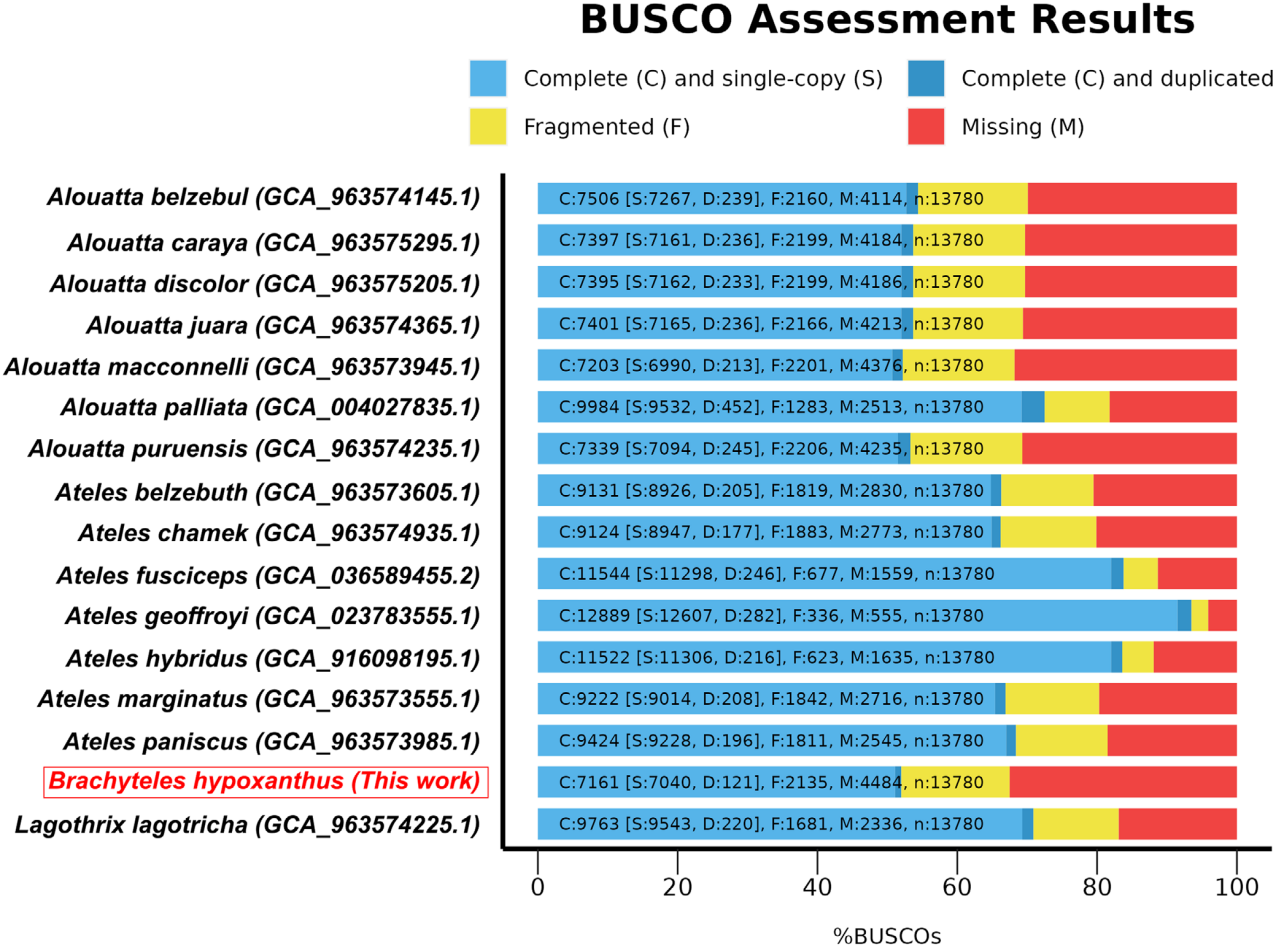
BUSCO completeness for the *Brachyteles hypoxanthus* genome assembled in this work and other 14 species of Atelidae with available genomes on NCBI.

A total of 610,819,479 raw sequencing reads were obtained for this HiSeq sequencing run. The assembly and scaffolding generated a draft genome with a total length of 2,518,664,220 bp distributed in 202,243 contigs (N50 = 29,134 bp), which ranged in size from 500 to 369,510 bp (Table 1). Our BUSCO completeness analyses revealed 52% of complete and 15.5% of fragmented genes (Figure 1, Table S3). The genome completeness in the family Atelidae ranges from 52% complete BUSCO genes in *B. hypoxanthus* to 93.5% in *Ateles geoffroyi* . Only one species has more than 90% of complete BUSCO genes, while seven species show BUSCO completeness ranging from 50% to 60% (Figure 1, Table S3). We highlight that the Atelidae genome projects are still in their early stages, with no genome yet available at the chromosomal scale. *Alouatta palliata* showed the largest genome assembled for Atelidae so far (3 Gb, Table S3), and presented a draft genome based on BUSCO completeness with 72.5% completeness and the highest duplicated gene copies on BUSCO (3.3%, Figure 1).

**TABLE 1:** Genome assembly statistics of the *Brachyteles hypoxanthus* draft genome.

Parameter	Value
Number of contigs	202,243
Number of contigs ≥ 1000 bp	163,404
Total length (bp)	2,518,664,220
Estimated genome size (bp)	2,483,403,561
Genome completeness	52%
Genome depth (times)	42.37
Largest contig (bp)	369,510
N50 (bp)	29,134
L50	24,277
GC (%)	40.70

**FIGURE 2:**
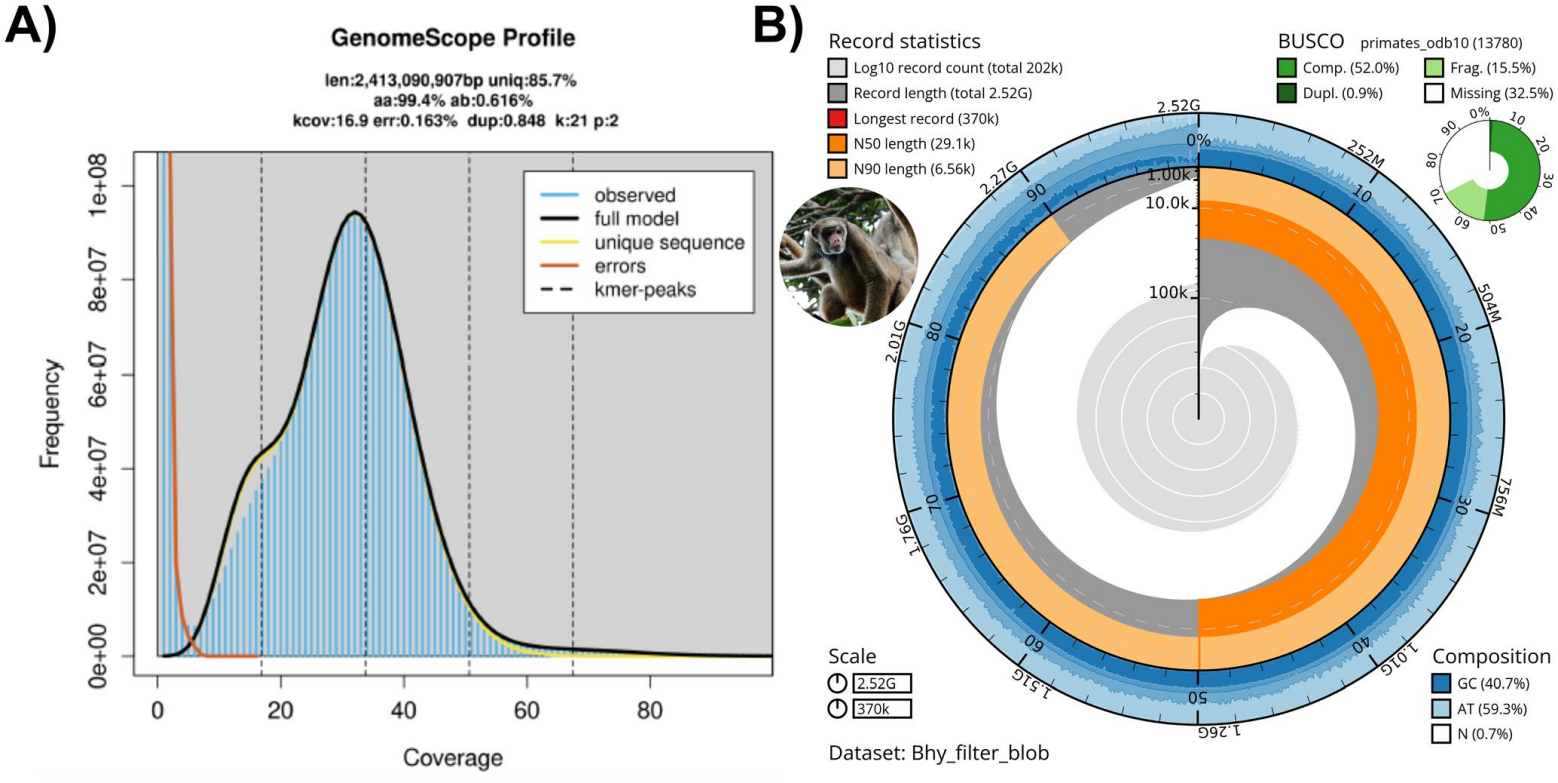
Genome size estimation and overall genome assembly metrics for *Brachyteles hypoxanthus* (A) Genome size profiles from 21‐mer analysis. The *X*‐axis represents the k‐mer depth and the *Y*‐axis represents the k‐mer frequency for a given depth. (B) The final genome assembly of the *B. hypoxanthus* metrics. The BlobToolKit Snailplot shows the BUSCO gene completeness and N50 metrics. The plot indicates a genome size of 2.52 Gb, and the longest contig obtained is 370 Kb. The plot also shows the N50 and N90 values, as well as the GC, AT, and N composition. A summary of the complete, duplicated, fragmented, and missing BUSCOs (primates_odb10) is shown on the right. The photo of the northern muriqui was taken by Ednardo Martins.

The estimated genome size was 2.4 Gb (heterozygous 0.62%), with about 12.3% being repeat sequences (Figure 2A). Among our contigs, only 10 contigs show similarity with another phylum (8 Arthropoda, 1 Ascomycota, and 1 Cnidaria) (Figure S1). These contigs were removed for the final assembly.

**FIGURE 3:**
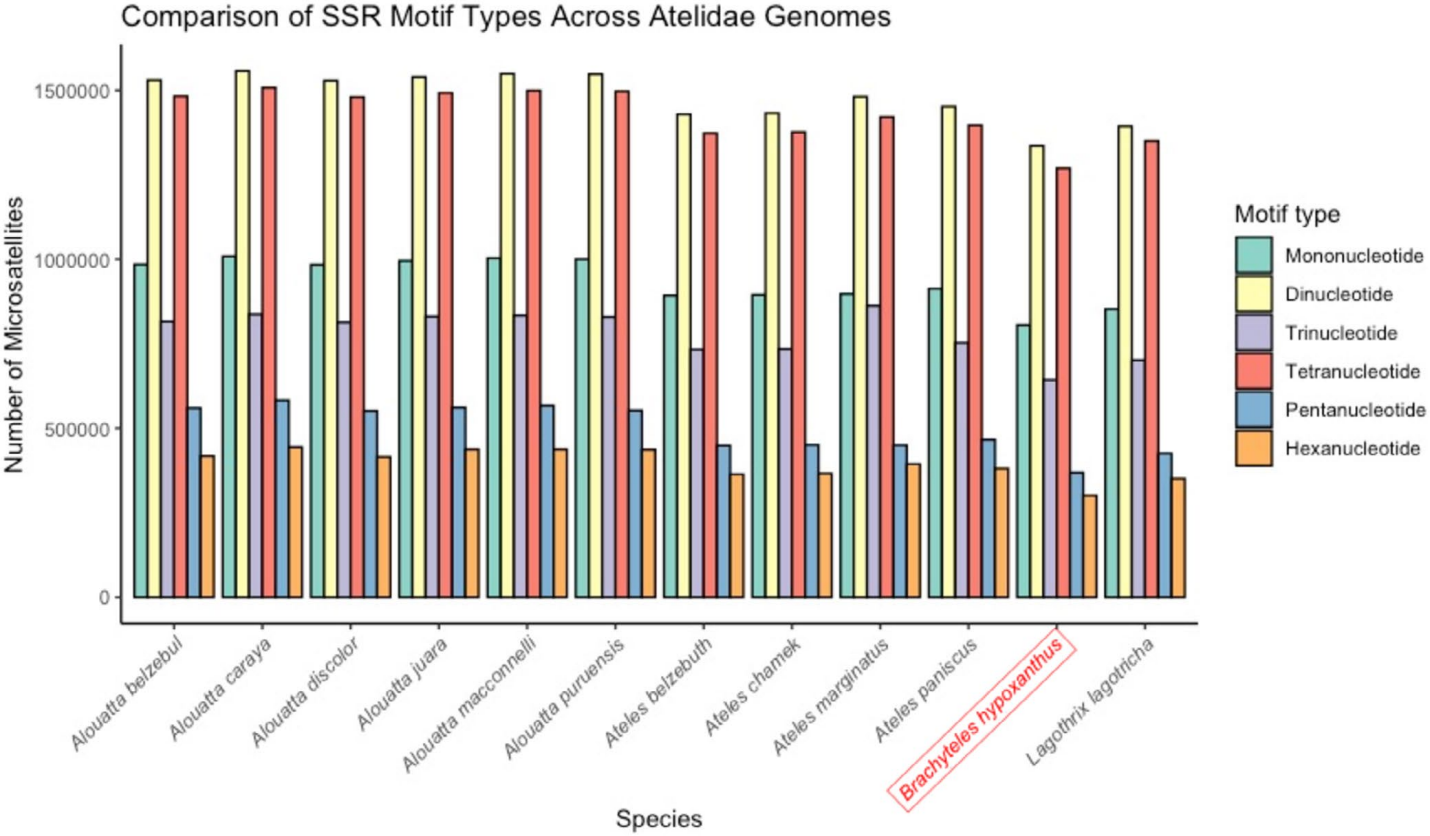
Number of microsatellite regions per motif type found in the Atelidae genomes.

We identified 4,722,442 microsatellite regions on the *B. hypoxanthus* genome assembly and an average of 5,521,895.33 microsatellite regions for all Atelidae genomes. In all Atelidae genomes analyzed here, we observed a constant pattern of dinucleotides being the most common motif type, followed by tetranucleotides and mononucleotides (Figure 3). The high number of microsatellite regions found in the *B. hypoxanthus* genome and in other Atelidae genomes may be related to its large genome size, as within primates, the abundance and density of SSR regions are significantly positively correlated with genome size (Song et al. 2021). Although mononucleotides were not the most abundant motif type in *B. hypoxanthus* and in the other Atelidae as they are in other primates, di‐ and tetranucleotides were also highly abundant as they are within the group (Song et al. 2021).

For primer design, focusing on assembled contigs larger than 10,000 bp and restricting the search to tetra‐ and pentanucleotide motifs with a minimum of 12 and 10 repeats, respectively, we were able to find 445 microsatellite regions that met our criteria. Of these, 343 were unique sequences and were used for primer design. Within the primer design criteria, 201 sequences ended up with primers, and of these, after the final filtering, 35 remained for primer in silico evaluation.

**FIGURE 4:**
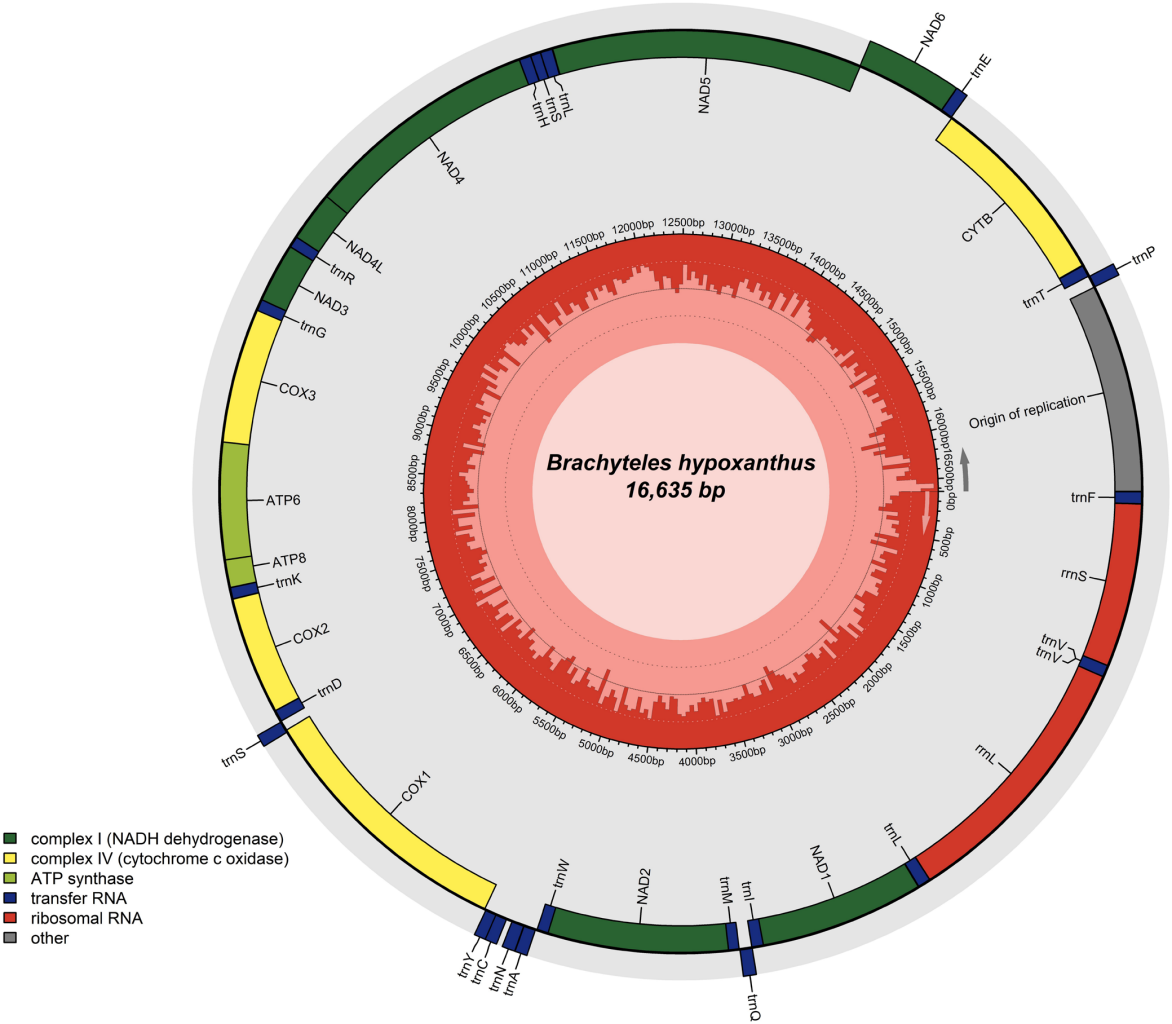
Graphical representation of the complete mitochondrial genome of *Brachyteles hypoxanthus* . Genes are colored indicated according to their functional classes, GC content is shown by the red bars inside the middle circle and reference position is indicated in base pairs (bp).

**FIGURE 5:**
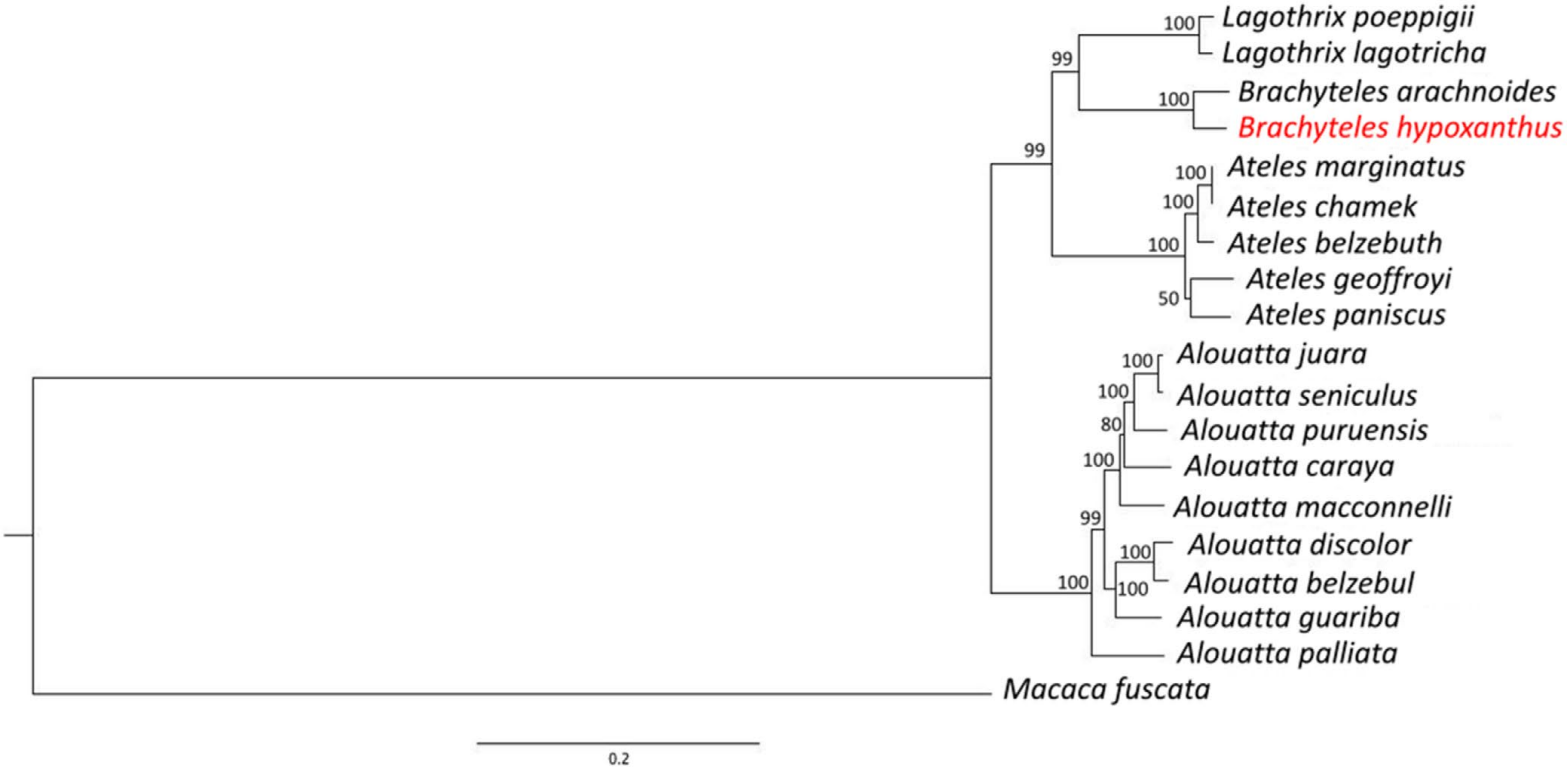
Maximum likelihood phylogenetic tree of the family Atelidae based on complete mitochondrial genome sequences. Values at the nodes represent bootstrap support from 1000 replicates. The mitochondrial genome of *Brachyteles hypoxanthus* highlighted in red was assembled on this work.

The complete mitochondrial genome of *B. hypoxanthus* showed a size of 16,635 bp with the expected structure of a mammalian mitochondrion and previously published Atelidae mitogenomes (da Fonseca et al. 2008; di Fiore et al. 2015; Janiak et al. 2022), consisting of 22 tRNAs, 2 rRNAs, 13 coding sequences (CDS), and an origin of replication (Figure 4). The phylogeny of Atelidae reconstructed with the available complete mtDNA sequences showed the genus *Brachyteles* to be more closely related to *Lagothrix* and *Ateles* than to *Alouatta* (Figure 5), as previously shown in phylogenies based only on mtDNA coding sequences and on nuclear data (di Fiore et al. 2015; Doyle et al. 2021). *Alouatta palliata* is placed as a sister taxon to all *Alouatta* species in agreement with results from a previous phylogeny for all Platyrrhini using mitochondrial genomes (Janiak et al. 2022) and other studies focused on the genus (Doyle et al. 2021; Povill et al. 2022; Villalobos et al. 2004). *Alouatta juara* appears closer to *A. seniculus* (Janiak et al. 2022) (Figure 5). In this tree topology, *A. belzebul* and *A. discolor* remain closely related but as separate lineages, as recently shown by cytochrome b (*cytB*) (Povill et al. 2022) and mitochondrial genome (Janiak et al. 2022) sequencing.

In the nucleotide diversity analysis of the mitogenome of the Atelidae species, the *cytB* gene presented one of the highest levels of nucleotide diversity for this genome (mean of π  = 0.137) (Figure S2A, Table S4). *cytB* has already been shown to be effective in distinguishing primate species by showing a large barcode gap between intra‐ and interspecific divergences (Nijman and Aliabadian 2010), and it is also an efficient barcode marker for the family Atelidae, as shown by this nucleotide diversity analysis and by how it has been used in evolutionary studies for taxonomic resolution purposes (di Fiore et al. 2015; Povill et al. 2022; Rylands and Mittermeier 2009). The mitochondrial hypervariable region, previously studied in other muriqui population genetic studies, now has primers for high‐throughput sequencing of ~199 bp to compare future sampling of the genus *Brachyteles* with the amplicons available in the database (Figure S2B). This tool will allow new phylogeographic analyses of muriqui populations newly sampled in the future with previously sequenced individuals, increasing sampling and genetic information on muriqui populations.

The 35 SSR primers, together with the two mtDNA primers (*cytB* and HVSI) and the two sex primers (AMEL and SRY), were included in the in silico evaluation. From the remaining 35 SSR primers, we excluded four that either had a self‐dimerization energy < −6 kcal/mol or a melting temperature lower than 48°C. The remaining 31 SSR primer pairs, together with the two mtDNA and two sex primers, were configured in multiplex sets based on their energy of cross‐dimerization estimated in openPrimeR (Table S5), while primers with energy of cross‐dimerization < −5 kcal/mol were placed in different multiplex sets (Table S5). The microsatellite regions selected after all filtering steps consisted mainly of tetranucleotides (28 loci), but there were also some pentanucleotide loci (3) (Table S5). Amplicon size varied from 151 to 250 bp, with a mean of 217.49 bp (Table S5), a size compatible with the read lengths of short‐read platforms. Multiplex sizes varied from 10 to 12 primers per multiplex set (Table S5), as smaller numbers of primers in a multiplex are preferred to increase the amplification success rate of non‐invasive samples (Trede et al. 2021). Increasing the number of multiplex sets to reduce the number of primers per set is not cost‐beneficial because it increases the cost of the PCR step, especially for non‐invasive sampling where the number of PCR replicates to confirm a genotype is high (Salado et al. 2021), and the amplification benefit is not significant (Trede et al. 2021).

Designing primer sets for multiplex PCR allows the genotyping of multiple loci in a single PCR reaction, a feature that can save time and money in population genetic studies with hundreds of samples while still providing reliable genotyping (Sint et al. 2012; Trede et al. 2021). Mixing SSR loci together with mtDNA loci and sex primers in a single high‐throughput sequencing run increases the genetic information generated by genotyping and sequencing all of these loci and allows various phylogeographic, population genetic structure and variability, and demographic questions to be answered with all of the data obtained. Very few species have such complete multiplex sets to amplify specific regions of their genomes (Donaldson et al. 2020; Šarhanová et al. 2018), and it represents a major advance in muriqui conservation at a reduced cost that can also be applied to other threatened species.

Regarding the draft genome, although we have achieved high sequencing coverage, we still have a considerably fragmented draft genome, and future sequencing studies using third‐generation sequencing may help to reduce this gap. However, for endangered species with no genomic data available and no specific genetic markers to study the population genetics of their wild populations, such as in the case of the northern muriqui, a new draft genome, a complete mitochondrial genome, and a set of microsatellite markers designed for the species that are now available will contribute to new evolutionary analyses of the group, as well as genetic tools to study and compare wild populations for conservation purposes. In addition, the fact that these primers have been specifically designed for genotyping by high‐throughput sequencing platforms with a focus on non‐invasive sample amplification will allow their use on a larger scale in terms of individuals and number of loci through non‐invasive sampling of unprecedented northern muriqui populations.

## Author Contributions

**Amanda Alves de Melo‐Ximenes:** conceptualization (equal), data curation (equal), formal analysis (lead), methodology (lead), writing – original draft (lead), writing – review and editing (lead). **Romina Batista:** data curation (equal), formal analysis (equal), methodology (equal), supervision (equal), writing – original draft (equal), writing – review and editing (equal). **Leonardo Carlos Jeronimo Corvalán:** conceptualization (equal), data curation (equal), formal analysis (equal), methodology (equal), writing – original draft (equal). **Tomas Marques‐Bonet:** data curation (equal), funding acquisition (equal), methodology (equal). **Lukas Kuderna:** data curation (equal), funding acquisition (equal), methodology (equal). **Kyle Farh:** data curation (equal), funding acquisition (equal), methodology (equal). **Jeffrey Rogers:** data curation (equal), funding acquisition (equal), methodology (equal). **Mariane da Cruz Kaizer:** data curation (equal), funding acquisition (equal), writing – review and editing (equal). **Jean Philippe Boubli:** data curation (equal), methodology (equal), project administration (equal), writing – review and editing (equal). **Fabiano Rodrigues de Melo:** data curation (equal), funding acquisition (equal), project administration (equal), writing – review and editing (equal). **Rhewter Nunes:** conceptualization (equal), supervision (equal), writing – review and editing (equal). **Mariana Pires de Campos Telles:** funding acquisition (equal), project administration (equal), supervision (equal), writing – review and editing (equal).

## Conflicts of Interest

The authors declare no conflicts of interest.

## Supporting information


**Figure S1.** BlopPlot plot of the *Brachyteles hypoxanthus* genome assembly. Each circle in the plot represents an assembly contig and the size of the circle is proportional to the length of the contig and the color is based on the taxonomic annotation identified based on its GC content and coverage and its legend is shown in the top right corner of the plot. The *x*‐axis represents the GC content and the *y*‐axis represents the read coverage. Histograms of the *x*‐ and *y*‐axis represent the contig distributions of GC content and coverage, respectively.

**Figure S2.** (A) Nucleotide diversity graph of complete Atelidae mitogenomes showing the cytochrome B gene (approximately between 14,500–16,000 bp) with high values of nucleotide diversity (π). The horizontal red line indicates 2× the median value of π as the cutoff for determining the regions of high nucleotide diversity. (B) Nucleotide diversity analysis of a small region of the mitochondrial hypervariable region of *Brachyteles* available on NCBI. The region between the vertical red lines represents the region selected for primer design for high‐throughput sequencing, which encompasses a region of high diversity among *Brachyteles* species.

**Table S1.** Species name, its respective GenBank accession number for complete mitochondrion genome sequence and which analysis it was included in this work. Phylogeny analysis corresponds to maximum likelihood tree for phylogeny reconstruction based on its complete mitochondrial sequence. Nucleotide diversity corresponds to analysis of nucleotide diversity for choosing more diverse regions to design primers in its flanking regions.

**Table S2.** NCBI Accession numbers for all hypervariable regions partial sequences of *Brachyteles* spp. sequences available on NCBI that were used for nucleotide diversity analysis and primer design.

**Table S3.** Comparative assembly statistics for all 15 Atelidae species with complete genome assemblies available on NCBI, including *Brachyteles hypoxanthus*, which was assembled in this work. All the metrics for the other species rather than *B. hypoxanthus* , except for the BUSCO completeness, were obtained from its NCBI accession submission information.

**Table S4.** Nucleotide diversity of Atelidae species mitochondrial genomes. Midpoints are positions in base pairs (bp) along the mitogenomes. Pi values represent nucleotide diversity values.

**Table S5.** Sets of microsatellite, mtDNA and sex primers for *Brachyteles hypoxanthus* for high‐throughput genotyping‐by‐sequencing. ^1^Primers previously developed by Di Fiore 2005. Tm: Melting temperature shown in “°C”.

## Data Availability

The draft of the nuclear genome and raw sequencing data are available under the BioProject ID PRJNA1201195 and the genome under the accession number JBLOJD000000000 on the National Center for Biotechnology Information (NCBI).
