# Supplementary figures and images for: First Assembly of a Draft Genome of the Critically Endangered Northern Muriqui ( Brachyteles hypoxanthus , Primates, Atelidae) Including Non‐Invasive Genotyping Strategies for the Species
Source: Ecol Evol. 2025 Aug 19;15(8):e71356. doi: 10.1002/ece3.71356 (PMC12365342; doi:10.1002/ece3.71356)

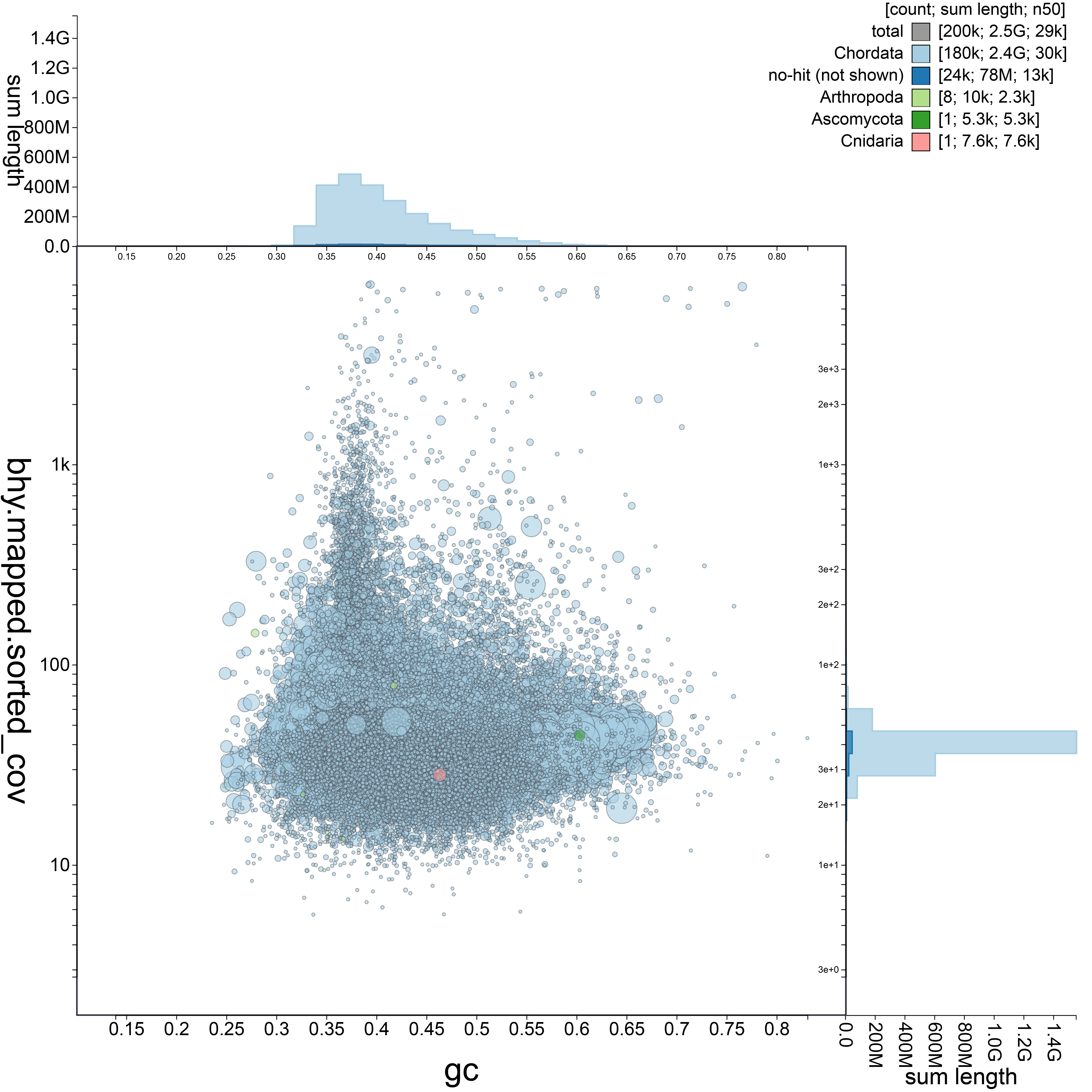

Supplement: Supplementary file 1 — Figure S1. BlopPlot plot of the Brachyteles hypoxanthus genome assembly. Each circle in the plot represents an assembly contig and the size of the circle is proportional to the length of the contig and the color is based on the taxonomic annotation identified based on its GC content and coverage and its legend is shown in the top right corner of the plot. The x‐axis represents the GC content and the y‐axis represents the read coverage. Histograms of the x‐ and y‐axis represent the contig distributions of GC content and coverage, respectively. [file ECE3-15-e71356-s005.jpg]

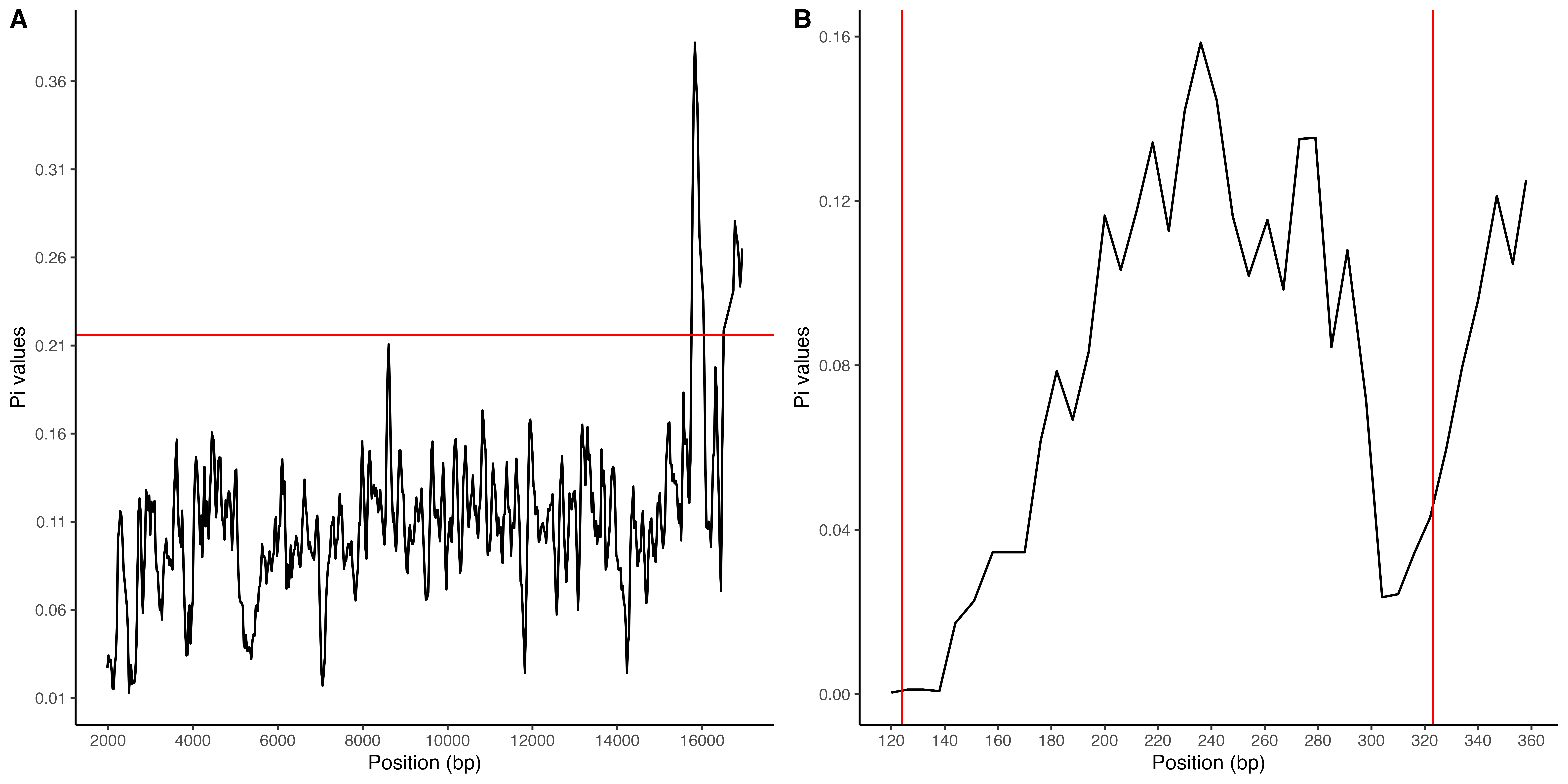

Supplement: Supplementary file 2 — Figure S2. (A) Nucleotide diversity graph of complete Atelidae mitogenomes showing the cytochrome B gene (approximately between 14,500–16,000 bp) with high values of nucleotide diversity (π). The horizontal red line indicates 2× the median value of π as the cutoff for determining the regions of high nucleotide diversity. (B) Nucleotide diversity analysis of a small region of the mitochondrial hypervariable region of Brachyteles available on NCBI. The region between the vertical red lines represents the region selected for primer design for high‐throughput sequencing, which encompasses a region of high diversity among Brachyteles species. [file ECE3-15-e71356-s007.jpg]
